# Development of Bonded/Riveted Steel Anchorages of Prestressed CFRP Strips for Concrete Strengthening

**DOI:** 10.3390/ma13102217

**Published:** 2020-05-12

**Authors:** Bartosz Piątek, Tomasz Siwowski, Jerzy Michałowski, Stanisław Błażewicz

**Affiliations:** 1 Department of Roads and Bridges, Rzeszow University of Technology, al. Powstancow Warszawy 12, 35-959 Rzeszow, Poland; siwowski@prz.edu.pl; 2 Institute of Nuclear Physics, Polish Academy of Sciences, ul. Radzikowskiego 152, 31-342 Cracow, Poland; jerzy.michalowski@ifj.edu.pl; 3 Department of Biomaterials and Composites, AGH University of Science and Technology, al. Mickiewicza 30, 30-059 Cracow, Poland; blazew@agh.edu.pl

**Keywords:** anchorage, CFRP strips, bonding, rivet, prestressing, strengthening system

## Abstract

CFRP (carbon fiber reinforced polymer) strips are currently often used to strengthen reinforced concrete structures in flexure. In order to ensure effective strengthening, proper connection between FRP material and concrete structure is needed. CFRP strips can be applied passively (only by bonding to the concrete surface) or actively (by prestressing before bonding). In the case of passive strengthening, CFRP strips connecting by bonding to the surface along the strengthened element are usually sufficient. However, active (prestressing) CFRP strips should be additionally anchored at their ends. Anchoring of unidirectional CFRP strips to the reinforced concrete is difficult because of their weak properties in transverse directions. The paper presents a development of mechanical steel anchorages used in an active CFRP flexural strengthening system for reinforced concrete structures. The anchorages were made of steel plates connected to CFRP strips with steel rivets and epoxy adhesive. They were developed within series of tests on specimens from small-scale to full-scale tested in an axial tensile scheme. The paper describes successive modifications of the anchorages as well as the results of full-scale tests. The final version of the anchorage developed during the research had a tensile failure force of 185 kN, which is sufficient value for CFRP strengthening purposes.

## 1. Introduction

The carbon fiber reinforced polymer (CFRP) materials are a viable alternative to structural strengthening and offer several advantages such as high strength-to-weight ratio, ease of transport and installation, excellent fatigue characteristics, and are noncorrosive thus highly durable. A few different approaches were investigated to assess the effectiveness of the CFRP materials for strengthening of civil structures. Since the beginning of the new millennium, hundreds of research papers on flexural strengthening of reinforced concrete (RC) structures with the CFRP materials have been published in scientific journals and conference proceedings. The most important research findings were reviewed and summarized in the following books [1,2,3,4] and state-of-the-art reports [5,6,7,8]. These reviews showed that the load-carrying capacity of the CFRP-strengthened beams can be significantly increased. In the case of the degraded beams, it was found out that the load-carrying capacity could be partly or fully restored by the CFRP strengthening system. On the basis of these and many more studies, it was concluded that using bonded CFRP materials as a flexural strengthening system can be effective.

By prestressing the CFRP strip, which combines benefits of passive bonded CFRP strips with the advantages due to external prestressing, the ultra-high tensile strength of carbon fiber composites can be utilized and much higher effectiveness of the strengthening technique is achieved. A greater CFRP tensile capacity was employed and it contributed to the load-carrying capacity of the strengthened structure under both service and ultimate conditions [9,10,11,12,13,14]. However, the anchor zones in strip-end locations were the main problem of beam strengthening with the CFRP prestressed strips. Without mechanical anchorages, the peeling failures at the strip ends were observed [15,16,17,18,19]. Premature debonding of the CFRP strips is one of the most common failure modes, and it could occur before the stresses in the CFRP material reach their tensile strength. Nevertheless, more detailed investigations are needed to obtain a good understanding of the way the forces acting inside the strengthening system and the way different parameters of the CFRP strip affect the behavior of the strengthened beam.

Over a dozen anchor systems have been developed for gripping and prestressing CFRP strips in recent years. The existing CFRP strip anchors can be divided into two major categories: Epoxy-based anchors and mechanical anchors (bolt-based or friction-based) [13,14,15,16,17,18,19]. Most of the existing CFRP strip anchors are epoxy-based or epoxy-and-friction-based anchors. In the latter group there are mechanical bolt-based anchors, mechanical bolt- and friction-based anchors, and mechanical friction-based anchors. It is evident that there is a wide range of different CFRP strip anchors available which are diverse in terms of materials used, geometry, strength, performance, design parameters, and failure modes. Anchors for smaller width (i.e., 15 mm) CFRP strips have a higher ultimate tensile strength than the anchors for wider (80–150 mm) strips. The epoxy-based anchors carry a small fraction (35–60%) of the ultimate tensile strength of the CFRP strip. The mechanical anchors carry a higher ultimate tensile load than the epoxy-based anchors. In addition, the epoxy-based anchors have a longer anchor length and installation time. Therefore, a mechanical anchor is the more efficient option. A majority of the existing anchors require unconventional manufacturing and complicated installation steps, and thus become highly expensive. The advantages and disadvantages of the design concepts of most anchors are reviewed in [19]. Although over a dozen CFRP plate anchors have been developed, very few are available commercially.

The new domestic approach in this field has been developed within R&D project at the Rzeszow University of Technology (RUT) [20]. The main elements of the first strengthening system (named Neoxe Prestressing System, NPS I) are special steel anchorages mounted on both ends of a single CFRP strip and a tensioning device, which uses a leverage effect to achieve the required prestressing force by means of a small hydraulic jack. However, the considerable limitation of this system was the maximum prestressing force of 85 kN, which was often too low for concrete strengthening purposes. Therefore, in the next research, the RUT developed a novel system (Neoxe Prestressing System II, NPS II) with the main goal to increase (to double, at least) the tensioning force, which could be applied during strengthening [21]. For this purpose, the new type of anchorages as well as a new tensioning device were developed. The paper describes briefly the NPS II strengthening system as well as the results of an anchor system examination. The tests proved a high efficiency of novel steel anchors as well as good effectiveness of prestressing and strengthening. The system has already been implemented on site on several RC bridges in Poland [22,23].

## 2. Description of Strengthening System

**Figure 1:**
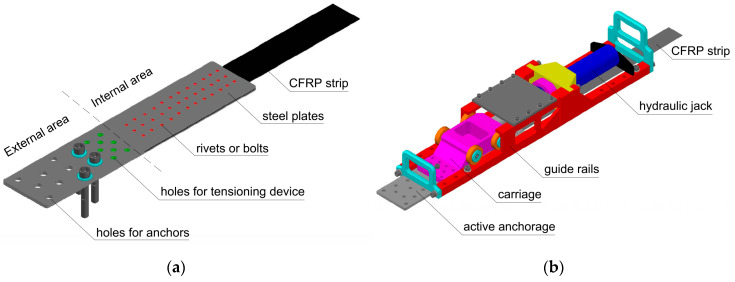
(**a**) Scheme of the active anchorage. (**b**) Scheme of the tensioning device.

The novel CFRP strengthening system (NPS II) consists of two main elements: Mechanical steel anchorages mounted on both ends of a single CFRP strip and a relevant tensioning device. Epoxy adhesive is used to bond a section of CFRP strip between anchorages to the concrete surface. The strips with determined length are delivered on site as ready-to-install, i.e., with two prefabricated steel anchorages mounted on both strip ends. There are two kinds of anchorages: An active anchorage combined with a tensioning device and a passive one, mounted on the second end of the CFRP strip. The anchorage has two functional areas: External and internal (Figure 1a). The strip is fixed within the internal area. The external area comprises holes for attaching the anchorage to the concrete surface by anchors and threaded holes for mounting the tensioning device (in the active anchorage only). The tensioning device is the second key element of the strengthening system. It has been developed using finite element analysis. The tensioning device consists of three separately installed components: Guide rails, carriage (bolted to the active anchorage), and hydraulic jack (Figure 1b). The hydraulic jack is driven by a manual pump and it can generate a maximum prestressing force of 170 kN. Thanks to the division of the device body into three small and lightweight parts (the heaviest element weighs 37 kg), its application on site is very fast and easy.

## 3. Development of Steel Anchorages

### 3.1. Materials

The strengthening system uses ultra-high-strength UHS 614 strips with cross-section of 1.4 mm × 60 mm with the ultimate tensile strength of 3200 MPa, modulus of elasticity of 160 GPa and the strain at failure of about 2% (obtained from the material tests). Larger-width strips can be applied as well, however, it would affect the results. The relevant comment is featured in Conclusions.

Steel plates of the anchorages are made of S355 steel grade with minimum tensile strength of 510 MPa, modulus of elasticity of 210 GPa, yield point of 355 MPa, and elongation of about 16%. CFRP strips are joined with steel plates by an epoxy-based adhesive as well as by 4-mm-diameter rivets made of S215 steel grade with minimum tensile strength of 375 MPa, modulus of elasticity of 210 GPa, yield point of 215 MPa, and elongation of about 25% (according to the steel grades).

Two types of the adhesives are used in the system. A standard epoxy-based adhesive is used for the final bonding of the strip to the concrete substrate between anchorages. The standard adhesive is characterized by flexural strength of 53.3 MPa, compressive strength of 107 MPa, flexural modulus of elasticity of 10.3 GPa, adhesion to dry concrete of 4.6 MPa, and glass transition temperature of 120 °C. It serves as a protective layer covering the strip between the anchorages. This type of adhesive is also used to bond CFRP strips with steel plates inside anchorages in the NPS I system (tested in S1N and S2N series). In the NPS II strengthening system, standard adhesive is used only to bond CFRP strips to a concrete surface while a modified epoxy adhesive with the addition of activated corundum sand is used in the anchorages. The modified adhesive is characterized by tensile strength of 32.5 MPa, tensile modulus of elasticity of 15 GPa, shear strength of 12 MPa, and glass transition temperature of 105 °C. The mechanical characteristics of the adhesives were obtained from the material tests.

### 3.2. Preliminary Study

The anchorages were developed within a series of tests on specimens from small scale to full scale. In the first step of the research, experiments on small-scale specimens were done in search of the most efficient steel/composite surface treatment method as well as optimal composition of epoxy adhesive. In the next step, the effectiveness of several types of steel-composite joints in medium-scale (1:2) were compared. These studies are described in details elsewhere [21]. Only the most important results of this part of the research are given here.

Based on the tests on small-scale specimens treated by various methods, the most efficient ones were determined to achieve efficient treatment of steel plates and CFRP strips before bonding them together. For steel plates the most effective method is sand blasting followed by cleaning with acetone, whereas, for CFRP strips, chemical cleaning using acetone-based solvent was found the best.

Medium-scale tests concerned several various types of steel-composite joints: Bonded, riveted, bolted, and hybrid bonded/bolted as well as bonded/riveted. The aim of this part of the research was to determine the most effective type of joint as well as to preliminarily define the characteristic parameters of the final full-scale specimens. The number and layout of the mechanical fasteners (bolts or rivets), the torque moment of the bolts, or clamping force of the rivets, as well as the joint length, were considered in medium-scale tests [21].

In the final step of the research, the two most efficient joining methods (bonded/bolted and bonded/riveted) were selected to test them finally on full-scale (1:1) specimens, i.e., actual anchorages. In this paper, the tests of full-scale specimens of bonded/riveted anchorages are described.

### 3.3. Full-Scale Tests

#### 3.3.1. Specimens

The single hybrid bonded/riveted (N-type) anchorage was made of three S355 steel plates: Two external with the dimensions of 550 mm × 128 mm and thickness of 2 mm or 3 mm, and one internal (spacer) with the dimensions of 249 mm × 126 mm and a thickness of 2 mm (comprising a 1.4-mm-thick composite strip and two layers of epoxy adhesive 2 × 0.3 mm). Steel plates were welded together along the edges to create a pocket, in which the CFRP strip end is fixed. The end of the strip was attached in the steel anchorage pocket along the section of approximately 300 mm (between external plates, within internal area of the anchorage (see Figure 1a)). The fastening of the strip was based on the use of an epoxy-based adhesive with fine sand (to obtain friction) and the set of steel rivets with a diameter of 4 mm and length of 10 mm. The anchorages transferred the tension force from the tensioning device to the strip by internal bonding, friction, and gripping, simultaneously. In order to prevent delamination of the CFRP strip in the areas of the rivets crossing the strip, carbon fabric overlays were used next to the steel plates’ edge.

#### 3.3.2. Modifications

**Figure 2:**
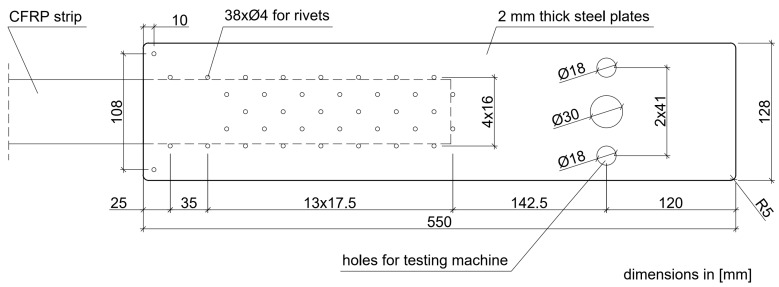
Arrangement of the rivets, S1N–S2N, series’ specimens (NPS I).

The tests of the N-type anchorages on full-scale specimens were carried out within five test series, S1N–S5N (three specimens in each series). In the first two series (S1N, S2N), the anchorages used in the first version of strengthening system (NPS I) were investigated for comparison with newly developed anchorages. The anchorages tested in S1N and S2N series were made of the 2-mm-thick external plates. A standard epoxy adhesive mix (the same which was used to bond CFRP strips to concrete surfaces along the strip between anchorages) was used to bond the steel plates to the CFRP strip. The steel plate and CFRP strip surfaces were not subjected to any special mechanical or chemical treatment prior to joining. The arrangement of rivets applied in these series is presented in Figure 2.

**Figure 3:**
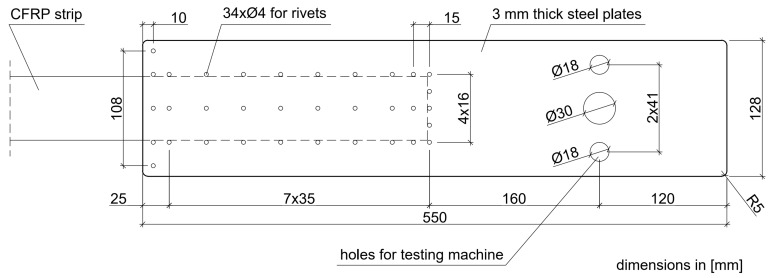
Arrangement of the rivets, S3N–S5N, series’ specimens (NPS II).

In the next three series (S3N–S5N), the anchorage specimens, made according to the modified technology (NPS II), were tested. The main modifications of the technology for anchorage specimen development consisted of increasing the thickness of the external plates to 3 mm, changing the number and arrangement of the rivets (Figure 3), using a new adhesive mix with parameters described in the Section 3.1., and preparing the surfaces before final bonding by sandblasting and chemical activation. The main differences between the anchorages of the NPS I system tested in S1N and S2N series and NPS II system tested in series S3N–S5N are summarized in Table 1.

The results obtained in the subsequent test series (S3N–S5N) and the observations of the anchorage specimens’ behavior under static load allowed successively introducing further technological modifications. The technology for anchorage development was, therefore, improved from test to test of subsequent series. A detailed description of the technology for specimen development in the subsequent test series is presented in the subsections below.

##### S1N Series

**Figure 4:**
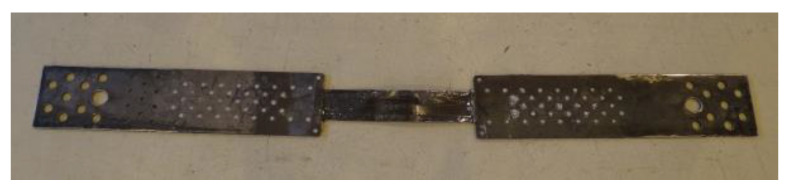
Double-sided N-type anchorage of the NPS I system tested in the S1N–S2N series.

In the first series, the anchorage specimens, made according to the technology used in the NPS I system, were tested. In this series, exceptionally high-modulus HM 614 strips with the ultimate tensile strength of 2730 MPa, the modulus of elasticity of 268 GPa, and the strain at failure of 1% were used (this type of strips was used in practice in the first version of the system, NPS I [20]). The specimens consisted of a section of CFRP strip with double-sided anchorage (Figure 4) and were made of the 2-mm-thick steel plates. In accordance with the technology used in the NPS I system, steel plates were not subjected to any mechanical or chemical treatment prior to bonding, apart from cleaning. The end parts of the strip (approx. 300 mm long), intended to be bonded between the steel plates, were matted with the sandpaper. For bonding, the standard epoxy adhesive was used in the following proportions: Component A (resin 61 g), component B (hardener 9.5 g). The adhesive bonding was heated at 110 °C for one hour. Then the bonding was strengthened with the rivets that were arranged, according to Figure 2. Riveting was made manually by an experienced technician after curing the adhesive layer.

##### S2N Series

In the second series of tests, the anchorage specimens, made according to the same technology as the S1N series, were tested. The only difference was to use the ultra-high-strength UHS 614 strips with parameters described in the Section 3.1. instead of HM 614 strips.

##### S3N Series

**Figure 5:**
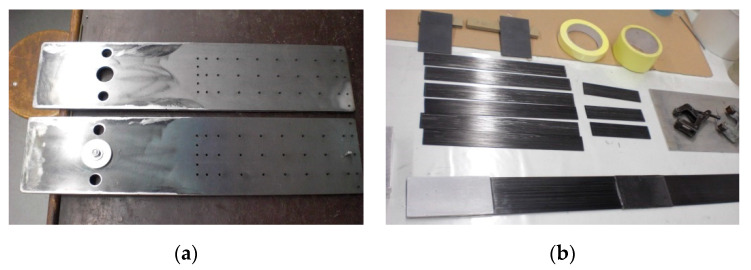
Elements of the steel plates (**a**) and CFRP strips (**b**) prepared for bonding.

**Figure 6:**
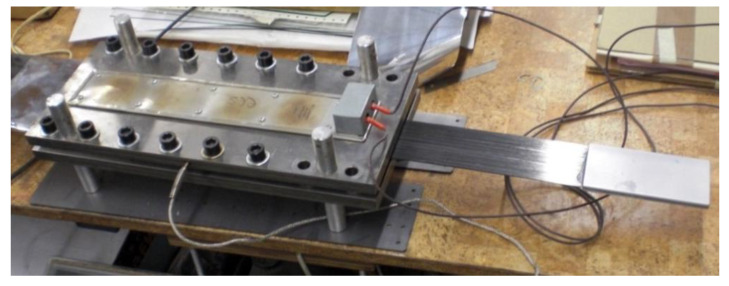
Curing of the adhesive bonding in a heating device.

**Figure 7:**
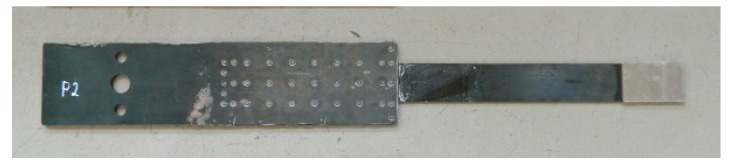
One-sided N-type anchorage of the NPS II system tested in the S3N-S5N series.

In the third series, the tests were conducted on the anchorage specimens, made according to the modified technology of bonding steel plates with the composite, developed on the basis of the results of adhesive tests carried out in the preliminary phase [21]. The external steel plate thickness was increased to 3 mm. The bonded surfaces were mechanically and chemically treated prior to bonding. The internal surfaces of the steel plate were sandblasted prior to welding (sand with the granulation of 0.2–1.0 mm). A new batch of sand was used each time for sandblasting to obtain a similar surface. The surface of the composite strips was prepared by matting with the sandpaper. In addition, the surfaces of both materials were chemically activated directly before bonding with a special solution based on the acetone. After preparing the steel plates and CFRP strips (Figure 5), the adhesive bonding was made. In order to do it, a modified adhesive composition with the addition of activated corundum sand was used, described in the Section 3.1. The adhesive bonding was subjected to curing at a reduced temperature of 80 °C for 3 h (Figure 6). The arrangement of the rivets was also changed, their number was reduced, and only one row of rivets crossing the composite strip was left (Figure 3). Two external rows of rivers are located outside the CFRP strip. The anchorage specimens were made as one-sided, and the free end of the strip was secured with the aluminum plates (Figure 7). The UHS 614 strips were used in the specimens of S3N and further series.

##### S4N Series

**Figure 8:**
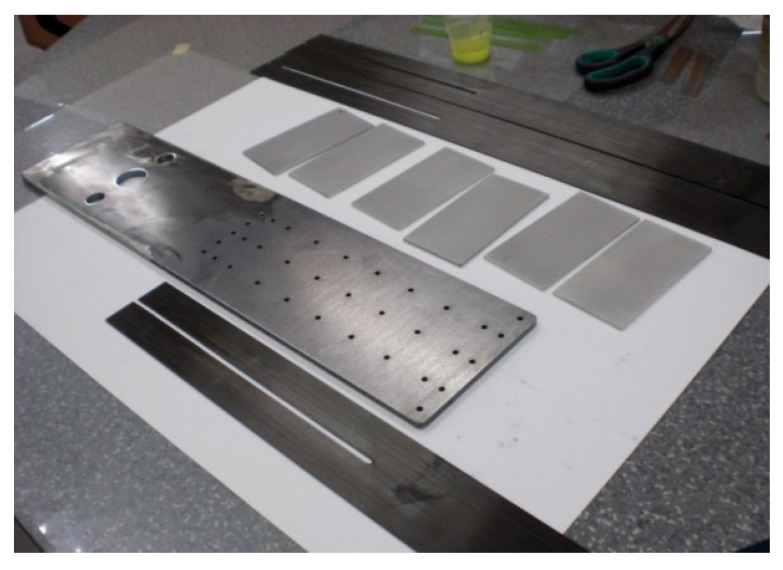
Elements of the S4N series’ specimens prepared for bonding (longitudinal cut in the strip).

The anchorage specimens tested in the S4N series were made in the same way as the S3N series specimens. The only difference was to make a longitudinal cut in the composite strip where the middle row of rivets was arranged (Figure 8).

##### S5N Series

**Figure 9:**
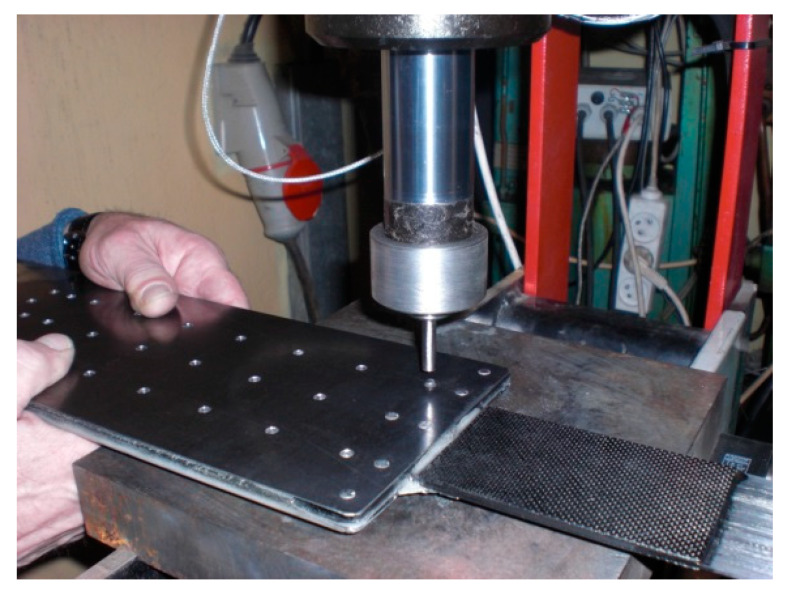
Rivet upsetting using the hydraulic press.

The specimens in the S5N series were prepared in the same way as for the S3N series (without longitudinal cut in the composite strip applied in S4N series) with the following changes. The curing time for the adhesive bonding was increased to 12 h. The rivets were tightened using a hydraulic press (Figure 9), by means of a constant upsetting force for each rivet. The pressure value on the pressure gauge amounted 100 atmospheres, which, calculated per the rivet cross-section, corresponded to the upsetting force of 28 kN. This force value ensured full rivet upsetting in the riveted hole. Exceeding this pressure value led to partial deformation of the anchorage plate.

#### 3.3.3. Test Method

**Figure 10:**
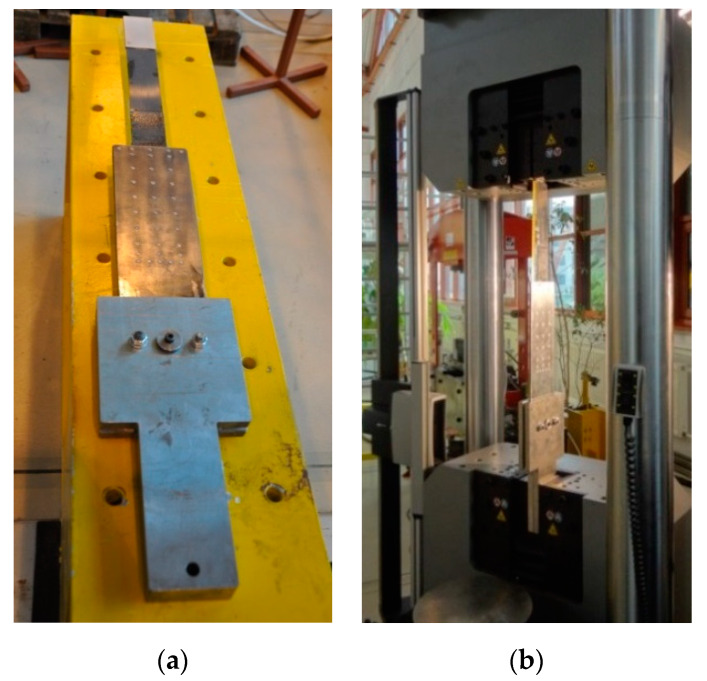
Anchorage specimen installed in the testing machine jaws (**a**) and the testing machine (**b**).

All the anchorage specimens in each series were subjected to the axial tensile tests in the Instron J1D 1200 kN testing machine (Instron, Norwood, MA, USA). The anchorages were installed in the machine by means of specially designed jaws allowing hinged attachment (Figure 10).

**Figure 11:**
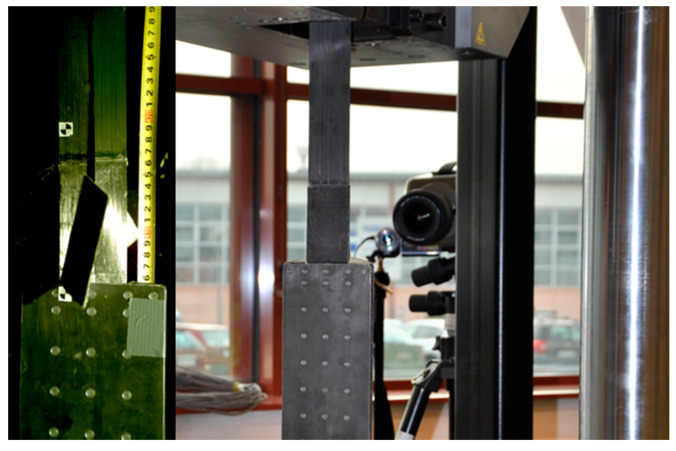
Recording the failure mode using the speed video camera with a high framing rate and an example of the view.

The tests were carried out with a displacement-controlled rate of 2 mm/min. During the tests, the increment of load and displacement between the jaws of the testing machine (displacement of the actuator piston) was measured. The measurement of these values was provided by the set of sensors built in the testing machine. Due to direct measurement through the sensors of the machine, presented displacements include slipping in the machine jaws, elongation of the CFRP strip, and displacements occurring in the joint itself. The specimens were tested until failure. The anchorage failure occurs by the rupture of the strip or failure of joint between CFRP strip and the steel plates. The failure mode of the anchorage specimens was recorded with the speed video camera Vision Research Phantom v640, which allowed recording over 2000 images per second (Figure 11).

## 4. Test Results

### 4.1. S1N Series

**Figure 12:**
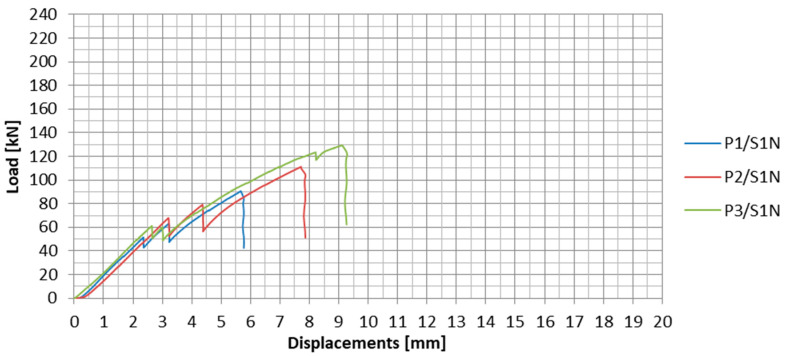
Load-displacement plots for all the specimens in the S1N series.

The load-displacement plots for the subsequent specimens tested in the S1N series are presented in Figure 12. The specimens are marked as PX/SYN, where X stands for the specimen number in the given series, and Y is the number of the test series. In Table 2, the values of failure loads and the achieved anchorage efficiencies for each specimen are summarized. The anchorage efficiency was determined as the ratio of the failure load of the anchorage specimen to the ultimate tensile strength (force) of the CFRP strip (in the case of S1N series, HM 614 was used with failure load equal to 229 kN obtained on the basis of material tests). The mean values of these quantities for the series as well as failure modes are given in the table.

During the first test series, the mean value of the failure load of the anchorage specimen was 110 kN. The standard deviation for the first series was 19.4 kN. The observed result dispersion exceeding 17% was quite significant. This may have been due to the high rigidity of the composite steel adhesive bonding and the associated high vulnerability to the heterogeneity of the steel-adhesive and adhesive-composite bonding.

On the load-displacement plots presented in Figure 12, sharp decreases of load, which represents partial slips of CFRP strip from the steel plates, can be observed. Two clear partial slips were observed during the tests for each anchorage specimen in series S1N. For the first two specimens, the first slip occurred at approx. 60% and the second at approx. 70% of the failure load (Table 3). In the case of the third specimen, both slips occurred one by one in a short time interval at less than 50% of the failure load. In addition, the third slip was observed at 123 kN, which occurred in the anchorage at the other end of the strip. The occurrence of these phenomena may indicate an uneven stress distribution in adhesive joints between both CFRP strip sides and external steel plates, as a consequence of the lack of their cooperation. With the difference of stresses, the increased tensile force caused local failure of the adhesive in one joint until the stresses in both joints were equalized. A greater dispersion of the subsequent slips in the tests of the first two specimens may indicate a higher uneven stress distribution in the adhesive joints at both sides of the CFRP strip.

**Figure 13:**
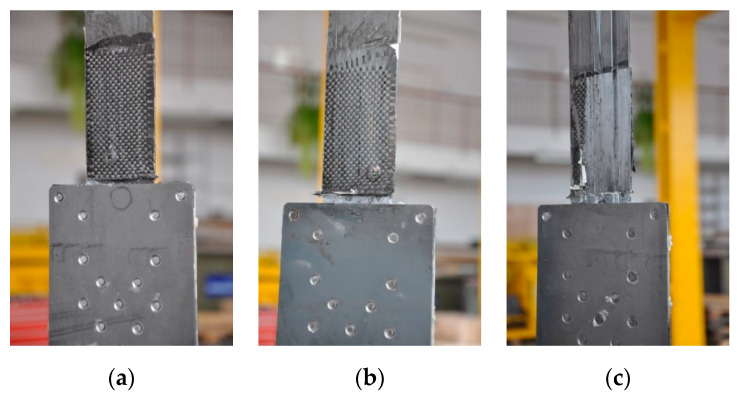
Failure mode of the subsequent specimens in the S1N series: (**a**) P1/S1N, (**b**) P2/S1N, (**c**) P3/S1N.

During the first series of tests, the failure mode of all three specimens was the same. The failure took place by the strips slipping out from the steel plates (Figure 13). Moreover, during the failure of the third specimen, there was chipping of a carbon fabric overlay, used to prevent delamination of the CFRP strip, spotted right next to the steel plate edge in the areas due to longitudinal crack of the strip (Figure 13c). The anchorage failure occurred suddenly and had a brittle character. There was no plastic phase in the behavior of the examined system.

### 4.2. S2N Series

**Figure 14:**
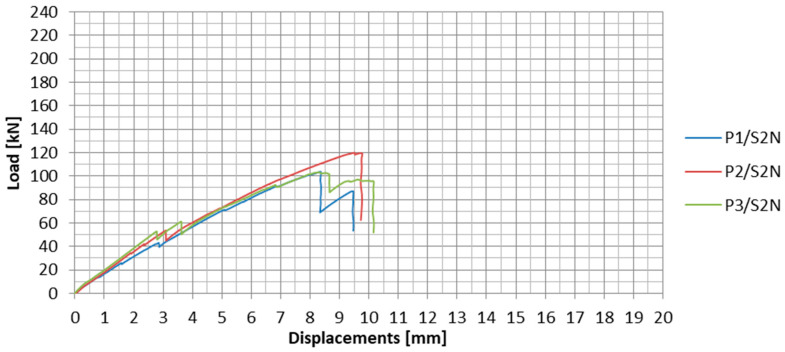
Load-displacement plots for all the specimens in the S2N series.

The load-displacement plots for the subsequent specimens from the S2N series are presented in Figure 14. In Table 4, the values of failure loads and the achieved anchorage efficiencies are summarized. The anchorage efficiency was determined as the ratio of the failure load of the anchorage specimen to the ultimate tensile strength (force) of the UHS 614 strip equal to 276 kN (value obtained on the basis of material tests).

In the second test series, the mean failure load of the specimen was 109 kN. The standard deviation value for the second series was 9.6 kN. The result dispersion was much lower than in the case of the first test series and did not exceed 9%. From both test series, it can be, therefore, concluded that the anchorages with the UHS 614 strips were less vulnerable to the heterogeneity of the steel-adhesive and adhesive-composite bonding. This may be due to the difference in the elasticity modules of the used CFRP strips.

During the tests of all the specimens, clear partial slips were observed at approx. 40–50% of the anchorage failure load, as well as several smaller slips occurring during the whole test. They were characterized by the strip partially sliding out from the steel anchorage. The partial slips are clearly visible in the load-displacement plots (Figure 14). The summary of the tensile forces resulting in the slips is presented in Table 5.

**Figure 15:**
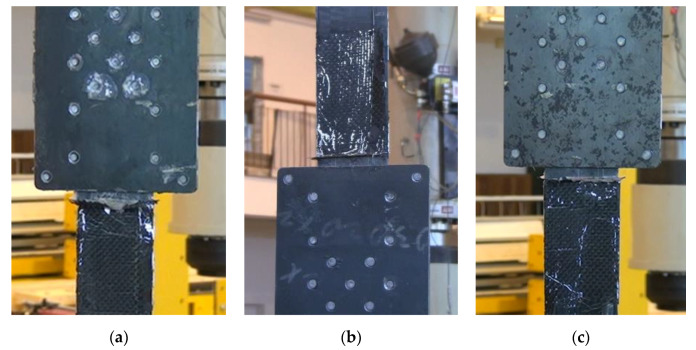
Failure mode of the subsequent specimens in the S2N series: (**a**) P1/S2N, (**b**) P2/S2N, (**c**) P3/S2N.

During the second test series, the failure mode of all three specimens was the same as in the S1N series. The failure took place by the strips slipping out from the steel plates (Figure 15).

### 4.3. S3N Series

**Figure 16:**
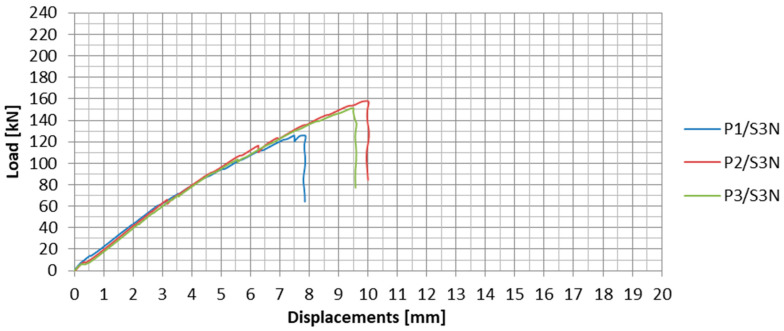
Load-displacement plots for all the specimens in the S3N series.

The load-displacement plots for the subsequent specimens from the S3N series are presented in Figure 16. In Table 6, the values of failure loads and anchorage efficiencies as well as description of failure modes are summarized. The anchorage efficiency was determined in the same way as in the S2N series.

During the third test series, in which the anchorages were made by means of the new technology (NPS II), the mean failure load was equal to 145 kN. The standard deviation for the third series was 16.7 kN. The observed result dispersion equaled 11.5%.

In the third test series, no apparent partial slips were observed. Only during the second specimen tests, two characteristic load decreases occurred in the plot (Figure 16): The first at 66.1 kN and the second at 116 kN. The strips were slipping out from the steel plates continuously throughout the entire test.

**Figure 17:**
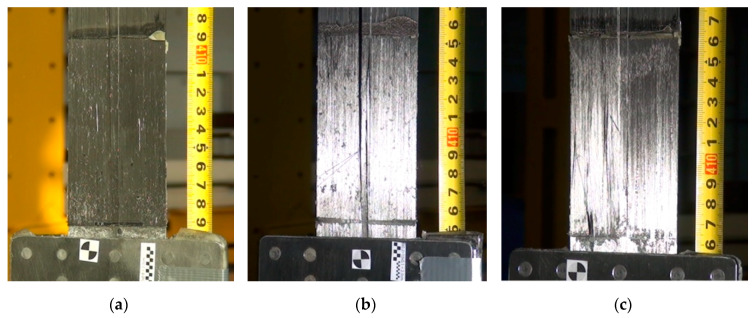
Failure mode of the subsequent specimens in the S3N series: (**a**) P1/S3N, (**b**) P2/S3N, (**c**) P3/S3N.

In this series, the failure mode took place by the strip slipping out from the steel plates accompanied by longitudinal cracks of the strip outside the anchorage along the middle row of the rivets and chipping of the overlay, used for strengthening the strips, next to the steel plate edge (Figure 17).

**Figure 18:**
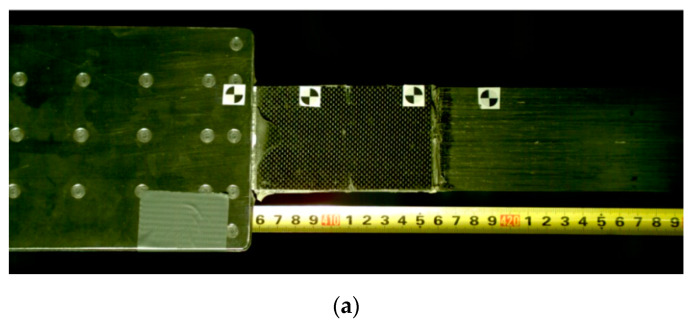

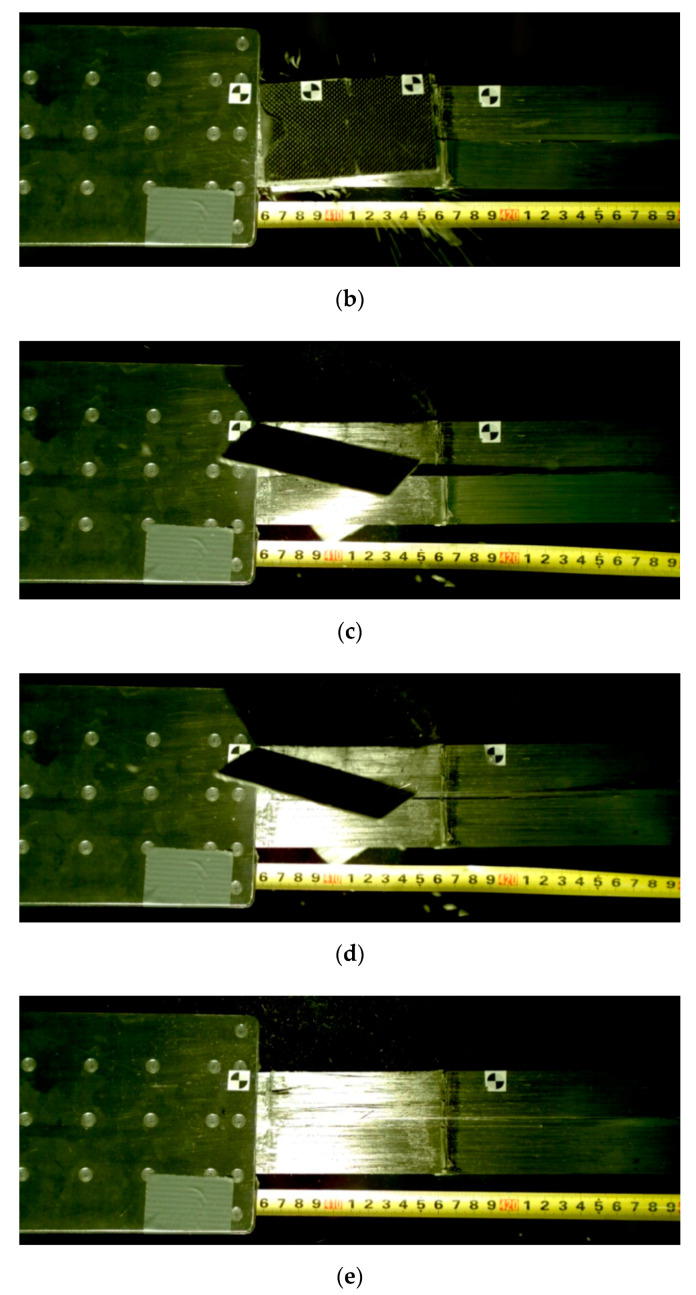
Sequence of failure of specimen P3/S3N: (**a**) Specimen directly before failure, (**b**) longitudinal shear cracking and bottom part of the strip slipping out, resulting in carbon fiber overlay delamination, (**c**) specimen directly before the upper part of the strip slipping out, (**d**) upper part of the strip slipping out, (**e**) specimen after failure.

The tests in the third series were recorded with the speed video camera. Thanks to that, it was possible to record the tests at the frame rate of 2300 frames per second, which allowed analyzing in detail the failure mode of the anchorages. The sequence of failure of exemplary specimen P3/S3N is presented in Figure 18.

The failure was initiated by longitudinal shear cracking of the strip along the middle row of the rivets and by the half of the strip sliding out from the anchorage (Figure 18b). This caused loosening of the strengthening carbon fiber overlays, which were bonded right next to edge of the steel plates (Figure 18b,c). In the final failure phase, the second half of the strip slipped out (Figure 18d). The entire failure mode, presented in Figure 18a–e, lasted about 0.01 s. A detailed analysis of the anchorage failure mode enabled introducing further modifications in the anchorage development technology.

### 4.4. S4N Series

**Figure 19:**
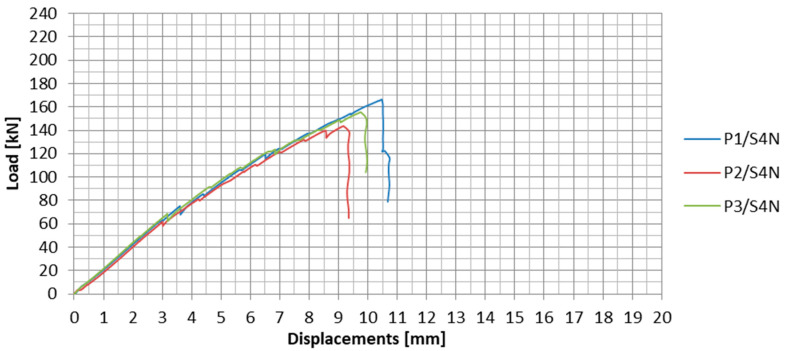
Load-displacement plots for all the specimens in the S4N series.

The load-displacement plots for the specimens tested in S4N series are presented in Figure 19 and the test results of this series are collected in Table 7.

During the fourth series of tests, the mean failure load value of the anchorage was 155 kN. The standard deviation for the fourth series was 11.4 kN and the variability coefficient equaled 7.3%.

**Figure 20:**
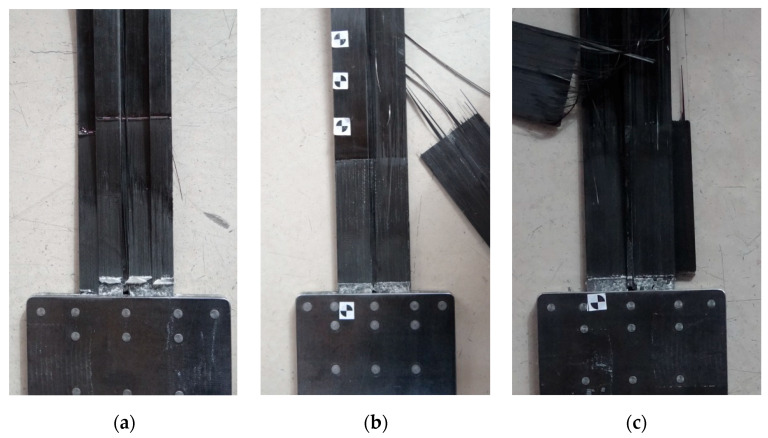
Failure mode of the subsequent specimens in the S4N series: (**a**) P1/S4N, (**b**) P2/S4N, (**c**) P3/S4N.

In the S4N series, the failure occurred similarly to that of the third series. The strips were slipping out from the steel plates and the longitudinal shear cracking was observed. In the case of the P2/S4N and P3/S4N specimens, there was one crack along the middle row of the rivets, which split the strip into two parts (Figure 20b,c), whereas in the case of the P1/S4N specimen, three cracks occurred splitting the strip into four fragments (Figure 20a). The specimen failure was also accompanied by chipping of the strengthening carbon fabric overlays. However, the overlays did not get completely delaminate from the strips, but after failure they remained bonded to the individual composite fibers, which indicated a better adhesion of the resin used to bond the strengthening overlays in this series of tests (Figure 20).

### 4.5. S5N Series

**Figure 21:**
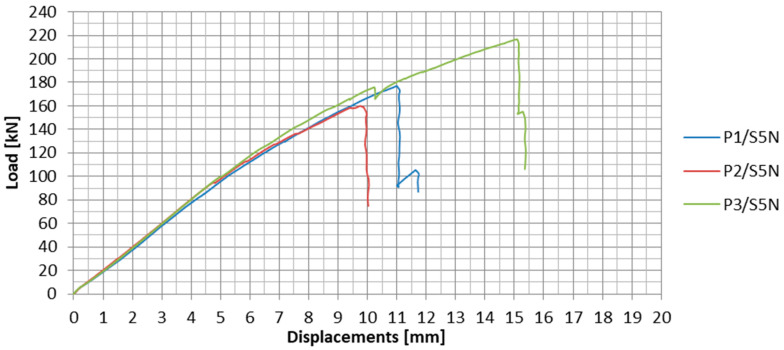
Load-displacement plots for all the specimens in the S5N series.

The load-displacement plots for the specimens tested in S5N series are presented in Figure 21 and the test results of this series are collected in Table 8.

During the fifth series of tests, the mean failure load value of the anchorage specimen amounted 185 kN. The mean anchorage efficiency equaled 67%, i.e., 11 percentage points more than in the fourth series. The resulting dispersion was also higher, mainly due to the P3/S5N specimen, in which a very high failure load of 217 kN was obtained. The standard deviation for the fifth series of tests was 29.2 kN and the variability coefficient equaled 15.8%.

**Figure 22:**
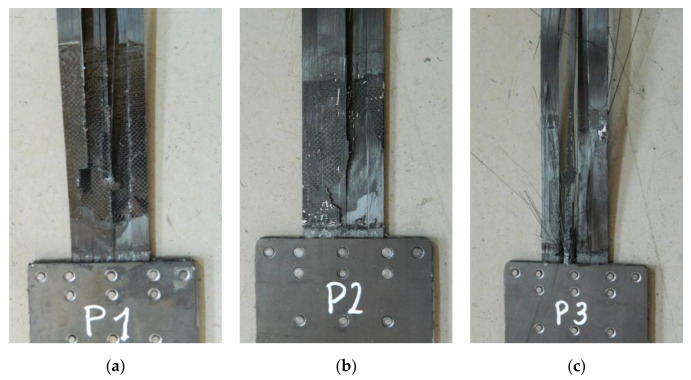
Failure mode of the subsequent specimens in the S5N series: (**a**) P1/S5N, (**b**) P2/S5N, (**c**) P3/S5N.

In the fifth series of tests, the failure mode also took place by the strip slipping out from the steel plates. As in the previous test series, longitudinal shear cracking of the strips occurred along the middle rivet row. In addition, there were cracks on both sides in approx. one-fourth and three-fourths of the strip width (Figure 22). In the P3/S5N specimen, which was characterized by the highest failure load (217 kN), a part of the strip was damaged by the rupture of carbon fibers (Figure 22c). In this series, the carbon overlays were not completely delaminated; parts of the overlays remained on the strip after failure (Figure 22).

## 5. Discussion

A resulting summary of the N-type anchorage tests is presented in Table 9. The mean failure load and anchorage efficiency, as well as the standard deviation and variability coefficient, were calculated for each series.

**Figure 23:**
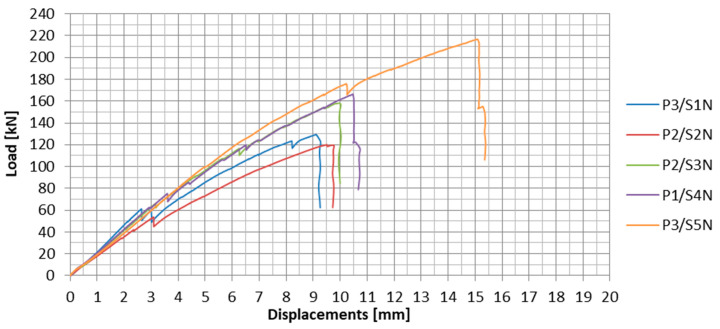
Load-displacement plots for the specimens with the maximum failure load in the subsequent series.

In Figure 23, the load-displacement plots were compared for the anchorage specimens for which the maximum failure loads were recorded in a subsequent series. In the first and fifth series of tests these were the P3 specimens, whereas in the second and third series these were the P2 specimens, and in the fourth series these were the P1 specimens.

**Figure 24:**
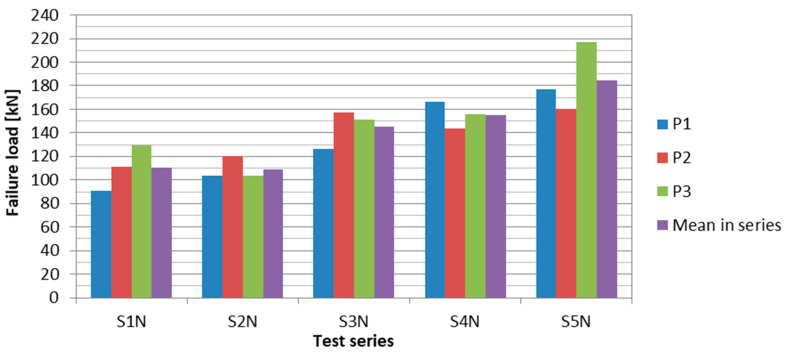
Increasing of failure loads in subsequent test series.

Both the maximum, minimum, and mean failure loads increased in the subsequent test series. Due to introducing further modifications in the anchorage development technology from series to series, increasingly higher load-carrying capacity of anchorages was achieved. This progress is illustrated in the plot in Figure 24.

Already in the third test series, in which the first modifications were introduced in the anchorage manufacturing technology, an increase of 33% in the mean failure load was obtained. The load-carrying capacity of the anchorages was improved largely due to using a new, modified adhesive composition and a special surface treatment of both connected materials. Changing the steel plate thickness and the rivet arrangement also influenced the increase of the anchorage load-carrying capacity. In the S4N series, due to the longitudinal cut in the composite strip where the middle row of rivets was arranged, there was an even greater increase of the mean failure load, by 42% compared to the S2N series. In addition, the highest homogeneity of the results was obtained in this test series. However, eventually, the idea of carrying out the cuts was abandoned due to the technological difficulties of such an operation. Instead, in the S5N series, the curing time of the adhesive bonding was extended from 3 to 12 h and rivet upsetting was controlled in the hydraulic press. This allowed to achieve a mean failure load of 185 kN, which indicated an increase of 70% compared to the original version of the anchorages (tested in S2N series). Moreover, in the P3/S5N specimen, an extremely high value of the failure load, i.e., 217 kN, was obtained. Thus, the composite material utility reached 80%. This indicated the great potential of this type of anchorages. In the last test series, however, a significant dispersion of results was noted (variability coefficient exceeding 15%). Therefore, the developed bonded/riveted anchorages require further studies on their improvement, especially in order to obtain better homogeneity of the results.

**Figure 25:**
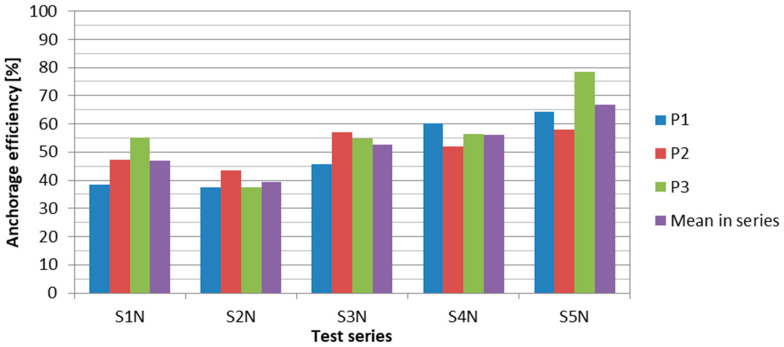
Anchorage efficiency in the subsequent test series.

The failure load was reflected in the anchorage efficiency, defined as the ratio of the failure load of the anchorage specimen to the ultimate tensile strength (force) of the CFRP strip (equal to 229 kN for HM 614 and 276 kN for UHS 614 strips). The applied modifications allowed to achieve an average anchorage efficiency of 67% in the last test series. This value was sufficient for a post-tensioning system for strengthening concrete structures because optimal strengthening effects are obtained with a strip prestressing level amounting to about 60% of CFRP tensile strength [24]. Figure 25 presents a comparison of the anchorage efficiency values in the subsequent test series.

Regardless of the test series, the failure always had a similar mode: The strips slipping out from the anchorage (steel plates) with simultaneous longitudinal shear cracking of the strips along the rivet row and the likelihood of an additional delamination in approx. one-quarter and three-quarters of the strip width. In the case of the S1N and S2N series (NPS I), the failure took place in two stages. At approximately 50% of the failure load, the sharp decreases of the load connected with the partial slips of the strip from the steel plates were observed. Thanks to introduced modifications, in the case of most specimens in the S3N–S5N series (NPS II), the partial slips were eliminated. The strip was gradually slipping out from the anchorage steel plates until failure.

## 6. Conclusions

The development of a novel anchorage for CFRP strengthening of RC beams was described. The anchorages were subjected to many tests in order to develop an optimum solution. Finally, anchorages for CFRP strips with cross-section of 60 mm × 1.4 mm with a tensile load-bearing capacity of 185 kN were developed and verified. Based on the test results of the N-type anchorages, the following conclusions can be drawn:

- Increased value of the N-type anchorage efficiency compared to the NPS I technology indicated the effectiveness of the proposed modifications.
- Rivets crossing the composite strip in the anchorage weakened its cross-section. A better solution is, therefore, to arrange the rivets in one row crossing the strip, which reduces the number of damaged fibers.
- Using the external steel plate with the thickness of 3 mm allowed for a more homogeneous distribution of stress after rivet upsetting. The 2-mm-thick steel plate used in the S1N and S2N series was too thin; strains occurring during rivet upsetting negatively affected the possibility to achieve a homogeneous compressive stress distribution in the strip.
- Lowering the temperature in the process of epoxy adhesive curing from 110 °C for the S1N and S2N series down to 80 °C in the S3N–S5N series increased the anchorage load-carrying capacity.
- Extending the adhesive bonding curing time to 12 h had a positive effect on the load-carrying capacity of the anchorages.
- Increasing the rivet arrangement density in the last two rows at the anchorage end of the strip and introducing the additional rivets on the edge perpendicular to the anchorage axis gave a positive result. The compensation occurred by pressing the nonlinear tensile stresses in the adhesive bonding, present in the internal part of the anchorage.
- The ultra-high-strength UHS 614 strip anchorages were less vulnerable to the heterogeneity of steel-adhesive and adhesive-composite joints compared to the high-modulus HM 614 strip anchorages.
- Further research should be carried out to increase the homogeneity of the obtained results and to increase the load-carrying capacity of the anchorage by fully incorporating the strip cross-section. The hybrid bonded/riveted anchorages have very high potential. The maximum failure load achieved so far amounted to 217 kN (79% of the CFRP tensile strength).

The system was developed particularly for strips of 60 mm in width because only these strips have been manufactured by the industry partner in the R&D project. Moreover, in domestic circumstances the 60-mm-wide CFRP strips are the most popular option for flexural strengthening. Larger width of the strips surely would affect the results [25,26]. The anchorage efficiency would be probably slightly lower for larger strips. The use of larger strips would require adjusting and modification of anchorage construction, in particular, the rivets’ arrangement. Some additional research would be necessary. However, it is believed that the idea of joining bonding, riveting, and friction in one anchorage could be efficient enough to be applied with larger strips.

Flexural strengthening of RC structures with prestressed CFRP strips is relatively new technology (about 15 years of real applications), so there are no proven life-cycle experiences with it. A few fatigue and accelerated long-term tests revealed that there is no danger to use this technology in the life-cycle term [27,28,29,30,31]. The decisive element of the technology is the durability of the adhesive used in CFRP strengthening systems, not exactly anchorages [32,33,34]. Moreover, it should be considered that the CFRP strengthening system is commonly used for existing structures, whose remaining life cycle is relatively short. Therefore, nowadays the use of CFRP prestressing systems are still growing worldwide. However, being aware of this shortcoming of the system, the research on the novel anchorage system comprised also fatigue and creep tests, which revealed the excellent long-term behavior of our anchorages (to be published elsewhere).

The novel strengthening system has been examined on the RC beams in the laboratory [21] as well as implemented to strengthen several real RC bridges [22,23]. The bridges are currently monitored in order to evaluate a long-term behavior of the system.

## 7. Patents

Patent No. 216773: “CFRP strip for strengthening building elements and device for tensioning CFRP strip before bonding it to a flat surface of a building element”, Polish Patent Office, Warsaw, Poland, 2014.

Patent Application No. P.417440: “Device for mounting CFRP strip to building elements and the method of mounting the strip to building elements”, Polish Patent Office, Warsaw, Poland, 2016.

## Figures and Tables

**Table 1:** Differences between anchorages used in the NPS I and NPS II systems.

System	Test Series	Steel Plate Thickness	Adhesive Composition	Surface Treatment	Rivets
NPS I	S1N–S2N	2 mm	standard	no treatment	38 ϕ 4 mm, arranged according to Figure 2
NPS II	S3N–S5N	3 mm	modified	sandblasting, chemical activator	34 ϕ 4 mm, arranged according to Figure 3

**Table 2:** Test results of the specimens tested in S1N series.

Specimen	Failure Load	Mean Failure Load	Anchorage Efficiency	Mean Anchorage Efficiency	Failure Mode
	(kN)	(kN)	(%)	(%)	-
P1/S1N	91	110	40	48	CFRP slip
P2/S1N	111		49		CFRP slip
P3/S1N	129		56		CFRP slip, longitudinal crack

**Table 3:** Summary of the loads resulting in the partial slip of the specimens tested in S1N series.

Specimen	Load at the First Partial Slip	Load at the First Partial Slip to Failure Load	Load at the Second Partial Slip	Load at the Second Partial Slip to Failure Load
	(kN)	(%)	(kN)	(%)
P1/S1N	51.3	57	63.1	70
P2/S1N	67.5	61	79.1	71
P3/S1N	61.2	47	60.2	47
Mean	60.0	55	67.5	62

**Table 4:** Test results of the specimens tested in S2N series.

Specimen	Failure Load	Mean Failure Load	Anchorage Efficiency	Mean Anchorage Efficiency	Failure Mode
	(kN)	(kN)	(%)	(%)	-
P1/S2N	103	109	37	39	CFRP slip
P2/S2N	120		43		CFRP slip
P3/S2N	103		37		CFRP slip

**Table 5:** Summary of the loads resulting in the partial slip of the specimens tested in S2N series.

Specimen	Load at the First Partial Slip	Load at the First Partial Slip to Failure Load	Load at the Second Partial Slip	Load at the Second Partial Slip to Failure Load
	(kN)	(%)	(kN)	(%)
P1/S1N	43.0	42	-	-
P2/S1N	53.5	45	-	-
P3/S1N	52.8	51	61.2	59
Mean	49.8	46	-	-

**Table 6:** Test results of the specimens tested in S3N series.

Specimen	Failure Load	Mean Failure Load	Anchorage Efficiency	Mean Anchorage Efficiency	Failure Mode
	(kN)	(kN)	(%)	(%)	-
P1/S3N	126	145	46	52	CFRP slip, longitudinal crack
P2/S3N	158		57		CFRP slip, longitudinal crack
P3/S3N	152		55		CFRP slip, longitudinal crack

**Table 7:** Test results of the specimens tested in S4N series.

Specimen	Failure Load	Mean Failure Load	Anchorage Efficiency	Mean Anchorage Efficiency	Failure Mode
	(kN)	(kN)	(%)	(%)	-
P1/S4N	166	155	60	56	CFRP slip, longitudinal crack
P2/S4N	144		52		CFRP slip, longitudinal crack
P3/S4N	156		56		CFRP slip, longitudinal crack

**Table 8:** Test results of the specimens tested in S5N series.

Specimen	Failure Load	Mean Failure Load	Anchorage Efficiency	Mean Anchorage Efficiency	Failure Mode
	(kN)	(kN)	(%)	(%)	-
P1/S5N	177	185	64	67	CFRP slip, longitudinal crack
P2/S5N	160		58		CFRP slip, longitudinal crack
P3/S5N	217		79		CFRP slip, partial rupture

**Table 9:** Summary of the test results in the subsequent test series.

Test Series	Mean Failure Load	Mean Anchorage Efficiency	Standard Deviation	Variability Coefficient
	(kN)	(%)	(kN)	(%)
S1N	110	47	19.4	17.5
S2N	109	39	9.6	8.8
S3N	145	52	16.7	11.5
S4N	155	56	11.4	7.3
S5N	185	67	29.2	15.8

## References

[1] Teng J.G., Chen J.F., Smith S.T., Lam L. (2002). FRP Strengthened RC Structures.

[2] Hollaway L.C., Teng J.G. (2008). Strengthening and Rehabilitation of Civil Infrastructures Using Fibre-Reinforced Polymer (FRP) Composites.

[3] Wu H.-C., Eamon C.D. (2017). Strengthening of Concrete Structures Using Fiber Reinforced Polymers (FRP): Design, Construction and Practical Applications.

[4] Kotynia R. (2019). FRP Composites for Flexural Strengthening of Concrete Structures: Theory, Testing, Design.

[5] Bakis C.E., Bank L.C., Brown V.L., Cosenza E., Davalos J.F., Lesko J.J., Machida A., Rizkalla S.H., Triantafillou T.C. (2002). Fiber-Reinforced Polymer Composites for Construction—State-of-the-Art Review. J. Compos. Constr..

[6] Mirmiran A. (2004). Bonded Repair and Retrofit of Concrete Structures Using FRP Composites.

[7] Taljsten B. (2004). FRP strengthening of concrete structures: New inventions and applications. Prog. Struct. Eng. Mater..

[8] Hollaway L.C. (2010). A Review of the Present and Future Utilisation of FRP Composites in the Civil Infrastructure with Reference to their Important In-service Properties. Constr. Build. Mater..

[9] Triantafillou T.C., Deskovic N., Deuring M. (1992). Strengthening of Concrete Structures with Prestressed Fiber Reinforced Plastic Sheets. ACI Struct. J..

[10] El-Hacha R., Wight R.G., Green M.F. (2001). Prestressed fibre-reinforced polymer laminates for strengthening structures. Prog. Struct. Eng. Mater..

[11] Nordin H., Täljsten B. (2006). Concrete Beams Strengthened with Prestressed Near Surface Mounted CFRP. J. Compos. Constr..

[12] Yang D.S., Park S.K., Neale K.W. (2009). Flexural behaviour of reinforced concrete beams strengthened with prestressed carbon composites. Comp. Struct..

[13] Aslam M., Shafigh P., Jumaat M.Z., Shah S.N.R. (2015). Strengthening of RC Beams Using Prestressed Fiber Reinforced Polymers—A Review. Constr. Build. Mater..

[14] Spadea G., Bencardino F., Sorrenti F., Swamy R.N. (2015). Structural effectiveness of FRP materials in strengthening RC beams. Eng. Struct..

[15] Garden H.N., Hollaway L.C. (1998). An experimental study of the influence of plate end anchorage of carbon fibre composite plates used to strengthen reinforced concrete beams. Compos. Struct..

[16] Pellegrino C., Modena C. (2009). Flexural Strengthening of Real-Scale RC and PRC Beams with End-Anchored Pretensioned FRP Laminates. ACI Struct. J..

[17] Michels J., Sena-Cruz J., Czaderski C., Motavalli M. (2013). Structural Strengthening with Prestressed CFRP Strips with Gradient Anchorage. J. Compos. Constr..

[18] Correia L., Teixeira T., Michels J., Almeida J.A.P.P., Sena-Cruz J. (2015). Flexural behaviour of RC slabs strengthened with prestressed CFRP strips using different-anchorage systems. Compos. Part B.

[19] Mohee F.M., Al-Mayah A., Plumtree A. (2016). Anchors for CFRP plates: State-of-the-art review and future potential. Compos. Part B.

[20] Siwowski T., Michalowski J., Blazewicz S. (2010). The new CFRP prestressing system for strengthening concrete structures. Inżynieria i Budownictwo.

[21] Piatek B., Siwowski T., Michalowski J., Blazewicz S. (2020). Flexural Strengthening of RC Beams with Prestressed CFRP Strips: Development of Novel Anchor and Tensioning System. J. Compos. Constr..

[22] Piątek B., Siwowski T. (2016). Investigation of strengthening effectiveness of reinforced concrete bridge with prestressed CFRP strips. Roads Bridges Drogi i Mosty.

[23] Siwowski T., Piątek B., Siwowska P., Wiater A. (2020). Development and implementation of CFRP post-tensioning system for bridge strengthening. Eng. Struct..

[24] Meier U. (1995). Strengthening of structures using carbon fibre/epoxy composites. Constr. Build. Mater..

[25] Breña S.F., Macri B.M. (2004). Effect of Carbon-Fiber-Reinforced Polymer Laminate Configuration on the Behavior of Strengthened Reinforced Concrete Beams. J. Compos. Constr..

[26] Tayfur S., Alver N., Tanarslan H.M., Ercan E. (2018). Identifying CFRP strip width influence on fracture of RC beams by acoustic emission. Constr. Build. Mater..

[27] Masoud S., Soudki K., Topper T. (2001). CFRP-Strengthened and Corroded RC Beams under Monotonic and Fatigue Loads. J. Compos. Constr..

[28] Heffernan P.J., Erki M.A. (2004). Fatigue Behavior of Reinforced Concrete Beams Strengthened with Carbon Fiber Reinforced Plastic Laminates. J. Compos. Constr..

[29] Rosenboom O., Rizkalla S. (2006). Behavior of Prestressed Concrete Strengthened with Various CFRP Systems Subjected to Fatigue Loading. J. Compos. Constr..

[30] Kotynia R., Walendziak R., Stoecklin I., Meier U. (2011). RC Slabs Strengthened with Prestressed and Gradually Anchored CFRP Strips under Monotonic and Cyclic Loading. J. Compos. Constr..

[31] Park H.B., Park J.-S., Kang J.-Y., Jung W.-T. (2019). Fatigue Behavior of Concrete Beam with Prestressed Near-Surface Mounted CFRP Reinforcement According to the Strength and Developed Length. Materials.

[32] You Y.C., Choi K.S., Kim J.H. (2012). An experimental investigation on flexural behavior of RC beams strengthened with prestressed CFRP strips using a durable anchorage system. Compos. Part B.

[33] Correia L., Sena-Cruz J., Michels J., França P., Pereira E., Escusa G. (2017). Durability of RC slabs strengthened with prestressed CFRP laminate strips under different environmental and loading conditions. Compos. Part B.

[34] Czaderski C., Meier U. (2018). EBR Strengthening Technique for Concrete, Long-Term Behaviour and Historical Survey. Polymers.

